# Shifting chemical defence or novel weapons? A review of defence traits in *Agarophyton vermiculophyllum* and other invasive seaweeds

**DOI:** 10.1007/s42995-021-00109-8

**Published:** 2021-07-05

**Authors:** Gaoge Wang, Yifei Ren, Shasha Wang, Minglei Hou, Florian Weinberger

**Affiliations:** 1 Institute of Evolution and Marine Biodiversity, College of Marine Life Sciences, Ocean University of China, Qingdao, 266003 China; 2 Marine Ecology Division, GEOMAR Helmholtz-Zentrum für Ozeanforschung Kiel, Düsternbrooker Weg 20, 24105 Kiel, Germany

**Keywords:** *Agarophyton vermiculophyllum*, Bioinvasion, Chemical defence, Novel weapons hypothesis, Shifting defence hypothesis

## Abstract

Seaweed bioinvasions increasingly affect coastal environments around the world, which increases the need for predictive models and mitigation strategies. The biotic interactions between seaweed invaders and invaded communities are often considered a key determinant of invasion success and failure and we here revise the current evidence that the capacity of seaweed invaders to deter enemies in newly reached environments correlates with their invasion success. Particularly efficient chemical defences have been described for several of the more problematic seaweed invaders during the last decades. However, confirmed cases in which seaweed invaders confronted un-adapted enemies in newly gained environments with deterrents that were absent from these environments prior to the invasion (so-called “novel weapons”) are scarce, although an increasing number of invasive and non-invasive seaweeds are screened for defence compounds. More evidence exists that seaweeds may adapt defence intensities to changing pressure by biological enemies in newly invaded habitats. However, most of this evidence of shifting defence was gathered with only one particular model seaweed, the Asia-endemic red alga *Agarophyton vermiculophyllum*, which is particularly accessible for direct comparisons of native and non-native populations in common garden experiments. *A. vermiculophyllum* interacts with consumers, epibionts and bacterial pathogens and in most of these interactions, non-native populations have rather gained than lost defensive capacity relative to native conspecifics. The increases in the few examined cases were due to an increased production of broad-spectrum deterrents and the relative scarcity of specialized deterrents perhaps reflects the circumstance that seaweed consumers and epibionts are overwhelmingly generalists.

## Introduction

As a component of global change and an ecological reflection of anthropogenic perturbation, seaweed invasions have received considerable interest from marine ecologists and biologists for more than a decade (Williams and Jennifer 2007). Introduced seaweeds have been detected in most marine ecoregions of the world and, in some places, they have generated drastic ecological impacts on coastal community structure, species abundance, richness, and functionality (Thomsen et al. 2012). A better understanding of the mechanisms that facilitate or inhibit invasions of exotic seaweeds is crucial for the assessment of incursion risks and for the identification of suitable management options (Richards et al. 2006; Schaffelke et al. 2006).

It is generally assumed that invasive species possess a set of traits that are relevant for the bioinvasion success and there are numerous hypotheses that aim to explain why certain species can become invasive (Chabrerie et al. 2019). An important subset of these is based upon the idea that on one hand, resident species in a community may reduce the success of introduced species if they can make use of them (for example as consumers or pathogens), while on the other hand, introduced species may experience a release from their coevolved enemies, which could then facilitate their survival in new environments (Elton 1958; Pearson et al. 2012). In the first case, invaders might be successful and selected if they are well defended against new enemies, whereas a loss of defensive capacity after invasion could be possible in the second case. In both cases, adaptations in the defence capacities of non-native populations relative to native populations are to be expected. A large body of studies—mostly conducted with terrestrial plants and their consumers—provides evidence of such adaptations, either toward weaker defence, if release from specialist consumers occurred, or toward stronger defence, if generalist consumers exert high feeding pressure on alien species (reviewed by Müller 2018). This led to the formulation of the “shifting defence hypothesis” (SDH, Doorduin and Vrieling 2011; Joshi and Vrieling 2005), which predicts that successful plant invaders should contain particularly high levels of defence compounds that deter generalist enemies, investing less into metabolites that deter specialist enemies. Another important hypothesis in this context is the “novel weapons hypothesis” (NWH, Callaway and Aschehoug 2000; Callaway and Ridenour 2004; Cappuccino and Carpenter 2005; Verhoeven et al. 2009), which predicts that exotic species should establish, proliferate and spread in new habitats if they own bioactive or deterrent metabolites to which the local species are not adapted. Similar to the SDH, the NWH is also mainly supported by studies conducted with terrestrial plants (Cappuccino and Arnason 2006; Donnelly et al. 2008; Inderjit et al. 2011; Jarchow and Cook 2009). Both hypotheses make predictions about the defensive capacity of successful bioinvaders and their offspring. In the case of the NWH, however, selection is predicted to favor those species that bring a new quality of defence traits to a habitat, whereas the SDH predicts selection of those species or individuals within a species whose quantitative allocation of resources is best adapted to the specific conditions of a newly gained habitat. Tests of both hypotheses therefore require somewhat different approaches. In the case of the NWH, tests can be conducted by screenings of invasive organisms for defence compounds that are absent from comparable native organisms in the invaded habitat. Rigorous tests of the SDH are often more challenging, since they require not only qualitative, but quantitative comparisons of defensive traits in native and non-native populations. Such traits are usually plastic and often dynamic and can potentially be affected by factors such as presence of enemies, availability of resources or environmental stress. It is for this reason that in most cases, transfer and acclimatization of specimens from different populations to a common environment is inevitable. Such common garden approaches are common in terrestrial plant invasion ecology but have so far been rarely realized with invasive aquatic organisms. In the following, we discuss the current evidence that the invasion success of seaweed invaders is influenced by biotic interactions, focusing on tests of NWH and SDH with algal models.

## Do seaweed invaders benefit from novel weapons?

As with terrestrial plants, macroalgae also have to cope with consumers and pathogens, and in addition with fouling organisms, such as bacteria, fungi, diatoms, invertebrates and macroalgae that constantly compete for settlement space and are often attracted by algal exudates and the polysaccharides on algal surfaces (Steinberg et al. 2002). To protect themselves, macroalgae have evolved defence mechanisms, which can be mechanical (e.g., carbonate skeletons for deterrence of consumers or epidermis shedding for removal of epiphytes) but are, in most cases, chemical and based on the production of deterrent or toxic primary or secondary metabolites. Most—if not all—seaweeds contain bioactive compounds, which makes them an increasingly frequent target in bioprospection (e.g., Freile-Peregrin and Tasdemir 2019; Wijesinghe and Jeon 2011). Also, in many invasive seaweeds, pronounced chemical defences have been detected, for example in *Grateloupia turuturu* and *Sargassum muticum* (Plouguerne ´ et al. 2008; Schwartz et al. 2017a, b), in *Asparagopsis taxiformis* (Greff et al. 2014), or in *Agarophyton vermiculophyllum* (Hammann et al. 2016a; Nylund et al. 2011; Rempt et al. 2012; Saha et al. 2016, 2017; Wang et al. 2017a, b). Defence-related compounds that have been identified in invasive macroalgae and were demonstrated to be ecologically relevant are listed in Table 1. Most of them—for example prostaglandins and eicosatetraenoids, phlorotannins or bromoform—are also present in large numbers of non-invasive species and are therefore not specific for invasive macroalgae. Correspondingly, cases supporting the NWH are scarcer among macroalgae than in terrestrial plants. The red alga *Bonnemaisonia hamifera* is the first marine invasive macroalga that has been shown to possess “novel” secondary metabolites that are associated with the adaption to new ranges and provide support of the NWH. *B. hamifera* has been introduced from East Asia to European coasts and is today one of the most conspicuous invasive red alga in Scandinavia (Thomsen et al. 2007). The compound 1,1,3,3-tetrabromo-2-heptanone is only found in *B. hamifera* and it strongly deters native herbivores in the invaded range (Enge et al. 2012). The compound also inhibits recruitment of native algal competitors (Svensson et al. 2013) and reduces bacterial densities (Nylund et al. 2008)*.*

**Table 1:** The identified compounds involved in chemical defences of invasive macroalga

Invasive species	Chemical compounds	Bioactivity	Range investigated	References
*Agarophyton vermiculophyllum*	Prostaglandins and other eicosatetraenoids	Anti-herbivore	Native and non-native	Hammann et al. (2016a, b), Nylund et al. (2011); Rempt et al. (2012)
*Asparagopsis taxiformis*	Mahorone and 5-bromomahorone	Antimicrobial activity against both marine and terrestrial microbes	Native	Greff et al. (2014)
*Asparagopsis armata*	Bromoform and dibromoaceticacid	Anti-microbial activity Anti-herbivore	Native	Paul et al. (2006a, b)
*Bonnemaisonia hamifera*	1,1,3,3-tetrabromo-2-heptanone	Anti-microbial activity Anti-herbivore	Non-native	Enge et al. (2012)
		Anti-competitor		Svensson et al. (2013)
*Fucus evanescens*	Phlorotannin	Anti-herbivore activity	Native and non-native	Wikström et al. (2006)
*Caulerpa racemosa*	Caulerpenyne	Anti-competitor activity	Non-native	Raniello et al. (2007)
*Caulerpa taxifolia*	Caulerpenyne and other terpenoids	Antimicrobial activity Anti-herbivore	Native and non-native	Amade and Lemee (1998); Guerriero et al. (1992); Paul and Fenical (1986); Ricci et al. (1999)
*Sargassum muticum*	Palmiticacid	Anti-diatom activity Anti-bacteria Inhibition of germination of *Ulva lactuca* spores	Non-native	Bazes et al. (2009)
*Sargassum muticum*	Phlorotannins	Low anti-bacteria anti diatom activity	Non-native	Kurr and Davies (2019)

Another long-standing example of an invasive macroalga that exhibits pronounced chemical defences is *Caulerpa taxifolia*, which originates from Australia and has invaded—among other areas—the Mediterranean Sea (Wiedenmann et al. 2001). Similar to its invasive congener *Caulerpa racemosa*, *C. taxifolia* produces Caulerpenyne, a toxic terpenoid that has been shown to affect consumers (Amade and Lemée 1998; Guerriero et al. 1992; Ricci et al. 1999) and even native competitors, such as the seagrass *Cymodocea nodosa* (Raniello et al. 2007). However, the native Mediterranean congener *Caulerpa prolifera* contains Caulerpenyne in similar amounts as *C. taxifolia* and *C. racemosa* (Jung et al. 2002). For this reason, Caulerpenyne is not a case supporting the NWH. Depending on environmental conditions, the strain of *C. taxifolia* that invaded the Mediterranean Sea can contain particularly high concentrations of Caulerpenyne (Amade and Lemée 1998), which could support the SDH. Yet, systematic comparisons of the Caulerpenyne content in individuals originating from native and non-native populations have, to the best of our knowledge, not been conducted.

## Evidence of shifting anti-herbivore defence and the metabolites involved

As outlined above, systematic testing of the SDH requires common garden experiments, for which representative sets of specimens from native and non-native populations need to be translocated to the same environment. Losses of single individuals may not occur during the transport, because these would represent a form of selection. Translocation without losses is often impossible with seaweeds, since they suffer from temperature change, anoxia or drought and lack dormant stages that are comparable to seeds. However, a protocol for loss-free transport has been established (Hammann et al. 2013) for the red macroalga *Agarophyton vermiculophyllum* (Ohmi) Gurgel, J.N. Norris and Fredericq (previously *Gracilaria vermiculophylla* (Ohmi) Papenfuss). *A. vermiculophyllum* is particularly resilient to transportation stress and perhaps for this reason is currently the most employed algal model in common garden experiments. Common garden experiments have revealed that invasive populations of *A. vermiculophyllum* are more resistant to temperature and salinity stress (Hammann et al. 2016b; Sotka et al. 2018) than native populations. As outlined in more detail below, such experiments also provided evidence that non-native and native *A. vermiculophyllum* populations differ in their resistance to certain consumers and to epibionts, including algal and animal settlers, as well as bacterial settlers.

**Fig. 1:**
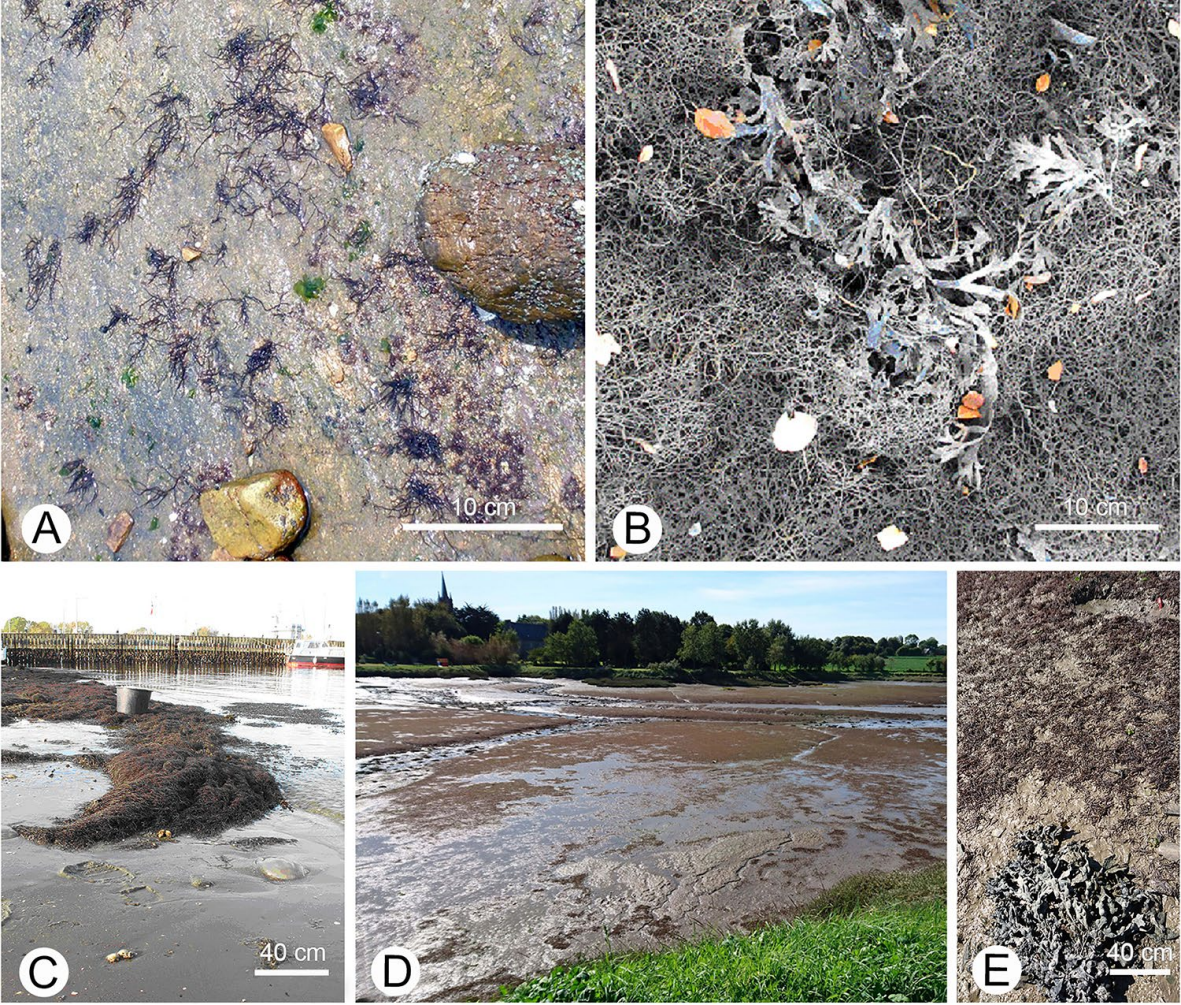
*A. vermiculophyllum* in native and non-native habitats. **A** Loose stands of small individuals attached to bedrock in a native habitat (Qingdao, China); **B** and **C** extensive unattached macroalgal mats of large individuals in a non-native habitat (Kiel, Germany); **D** and **E** extended dense stands of smaller individuals that are anchored in soft muddy substratum (Pouldouran, France)

*A. vermiculophyllum* was formerly endemic to East Asia (Ohmi 1956) but has successfully invaded numerous North American and European coastal habitats over the past 3–4 decades (Bellorin et al. 2004; Nettelton et al. 2013; Rueness 2005; Sfriso et al. 2010; Thomsen et al. 2006; Weinberger et al. 2008). After arrival in a new environment, a species typically establishes itself as abundant entangled mats or expansive drifting blooms in coastal lagoons and estuaries (Thomsen et al. 2007), which have the potential for a massive influence on coastal systems (reviewed by Hu and Juan-Bautista 2014) (Fig. 1). The interactions of invasive *A. vermiculophyllum* populations with consumers in the new habitats have frequently been studied (e.g., Cacabelos et al. 2010; Engelen et al. 2011; Nejrup and Pedersen 2012; Weinberger et al. 2008). Weinberger et al. (2008) found that in the Baltic Sea mesograzers (the isopod *Idotea balthica*, the periwinkle *Littorina littorea* and amphipods of the genus *Gammarus*) avoided feeding on *A. vermiculophyllum* and preferred the native species *Fucus vesiculosus*. Vinzent (2009) obtained similar results in laboratory feeding studies with the same three mesograzers, but increased numbers of native algal species, mimicking the algal assemblage in the natural environment. He found that all three consumers preferred fast-growing macroalgae (*Ulva* sp. and *Ceramium virgatum* Roth, formerly *C. rubrum*) to *A. vermiculophyllum* and *F. vesiculosus* when given a choice. Feeding rates were low when only *A. vermiculophyllum* was offered and the herbivores grew less well than when fed with other native algae. Nejrup and Pedersen (2010) reported that grazing on *A. vermiculophyllum* was also very low in Baltic Sea habitats. Furthermore, Nejrup and Pedersen (2012) compared the deterrence of herbivores by *A. vermiculophyllum*, *F. vesiculosus*, *C. virgatum* (formerly *C. rubrum*), and *U. intestinalis* (formerly *Enteromorpha intestinalis*), using three common consumers (*I. baltica*, *Gammarus locusta* and *L. littorea*) in no-, two- and multiple-choice trials. Together, the results showed that grazers avoided *A. vermiculophyllum* whenever there was a choice. Hammann et al. (2013) used the common garden method to investigate differences in the palatability to consumers between six native (Chinese and Korean) and eight non-native (European and Mexican Pacific coast) *A. vermiculophyllum* populations. The authors conducted repeated feeding assays with specimens from all fourteen populations, both in their native range at Qingdao in China and in the non-native range at Kiel in Germany. The consumers in these experiments were periwinkles (*Littorina brevicula* in China and *L. littorea* in Germany). Hammann et al. (2013) observed that the native feeding-enemy *L. brevicula* generally ate more *A. vermiculophyllum* than the non-native *L. littorea*, while both periwinkles consumed less of the non-native individuals than of the native ones. Such differences in palatability could hint either at higher concentrated feeding cues in native individuals or at higher concentrated deterrents in non-native individuals. A limiting factor, and for this reason, an important feeding cue for *Littorina,* is protein (van Alstyne et al. 2019). However, Hammann et al. (2013) observed no significant correlation between C:N ratios and biomass consumption by the two types of snails and suggested that the observed difference in palatability was due to different deterrence. Comparing native populations, the authors observed particularly low palatability in one Korean population that had previously been identified as being particularly genetically similar with all the non-native populations of *A. vermiculophyllum* (Gulbransen and McGlathery 2012; Kim et al. 2010). Based on these findings, the authors proposed that genotypes with particularly strong anti-herbivory traits may have been selected and facilitated the invasion success of *A. vermiculophyllum*. However, later studies revealed that the donor region of non-native *A. vermiculophyllum* populations is probably located in northeast of Japan and not in Korea (Krueger-Hadfield et al. 2017)*.*

To elucidate the mechanisms behind the anti-herbivore defence of *A. vermiculophyllum* observed both in laboratory and field studies, metabolomics, in combination with artificial food bioassays, were used. Nylund et al. (2011) found that both direct grazing by the generalist mesograzer *I. baltica* and simulation of herbivory by mechanical wounding could cause similar metabolic responses. The most pronounced metabolic change after simulation of herbivory was an upregulation of arachidonic acid-derived oxylipins. Arachidonic acid (AA) is the precursor of prostaglandins and some other bioactive compounds and accounts for up to 45% of the total content of fatty acids in *A. vermiculophyllum* (Imbs et al. 2012; Sajiki 1997). AA can be rapidly activated after wounding treatment, and immediate downstream reactions result in the production of prostaglandins (PG), hydroxylated fatty acids and AA-derived conjugated lactones (Nylund et al. 2011; Rempt et al. 2012). Feeding bioassays conducted with PGA_2_ and hydroxylated eicosatetraenoic acids have repeatedly shown that these compounds can indeed deter different crustacean and gastropod mesograzers—including periwinkles and isopods—at physiologically relevant concentrations (Hammann et al. 2016a, b; Nylund et al. 2011; Rempt et al. 2012), which strongly suggests that their wound-activated production may be an important mechanism of anti-herbivore defence in *A. vermiculophyllum*.

Correspondingly, Hammann et al. (2016a, b) observed that the wound-activated metabolites 15-keto-PGE_2_, PGE_2_, PGA_2_ and 7,8-di-hydroxy-eicosatetraenoic acid are significantly more concentrated in non-native populations than in native populations. Thus, the observed differences in palatability between native and non-native populations of *A. vermiculophyllum* to periwinkles (Hammann et al. 2013) can be explained with an upward-shift in the production of AA-derived defence compounds during or after invasion. Another common garden study compared the palatability of *A. vermiculophyllum* originating from 14 native Japanese and 25 non-native European and North-American sites to the amphipod consumer *Amphitoe valida* and found no evidence of shifting defence, as the consumer exhibited a non-significant preference for non-native individuals (Bippus et al. 2018). This discrepancy could either hint at a contrasting sensitivity of *A. valida* compared to periwinkles and isopods or result from the fact that Hammann et al. (2013) and Bippus et al. (2018) tested different populations in the native range.

Several other studies that were conducted with other invasive macroalgae (e.g., Schwartz et al. 2017b; Wikström et al. 2006) and simply compared field-collected specimens that were not previously acclimatized to common gardens also found increased palatability in non-native compared to native populations. Given that most marine herbivores are generalist rather than specialist feeders (Hay and Steinberg 1992) a strong defensive capacity against herbivores based upon broad-range deterrents might indeed facilitate invasion success in many habitats.

## Evidence of shifting defence against microalgal and macroalgal settlers

**Fig. 2:**
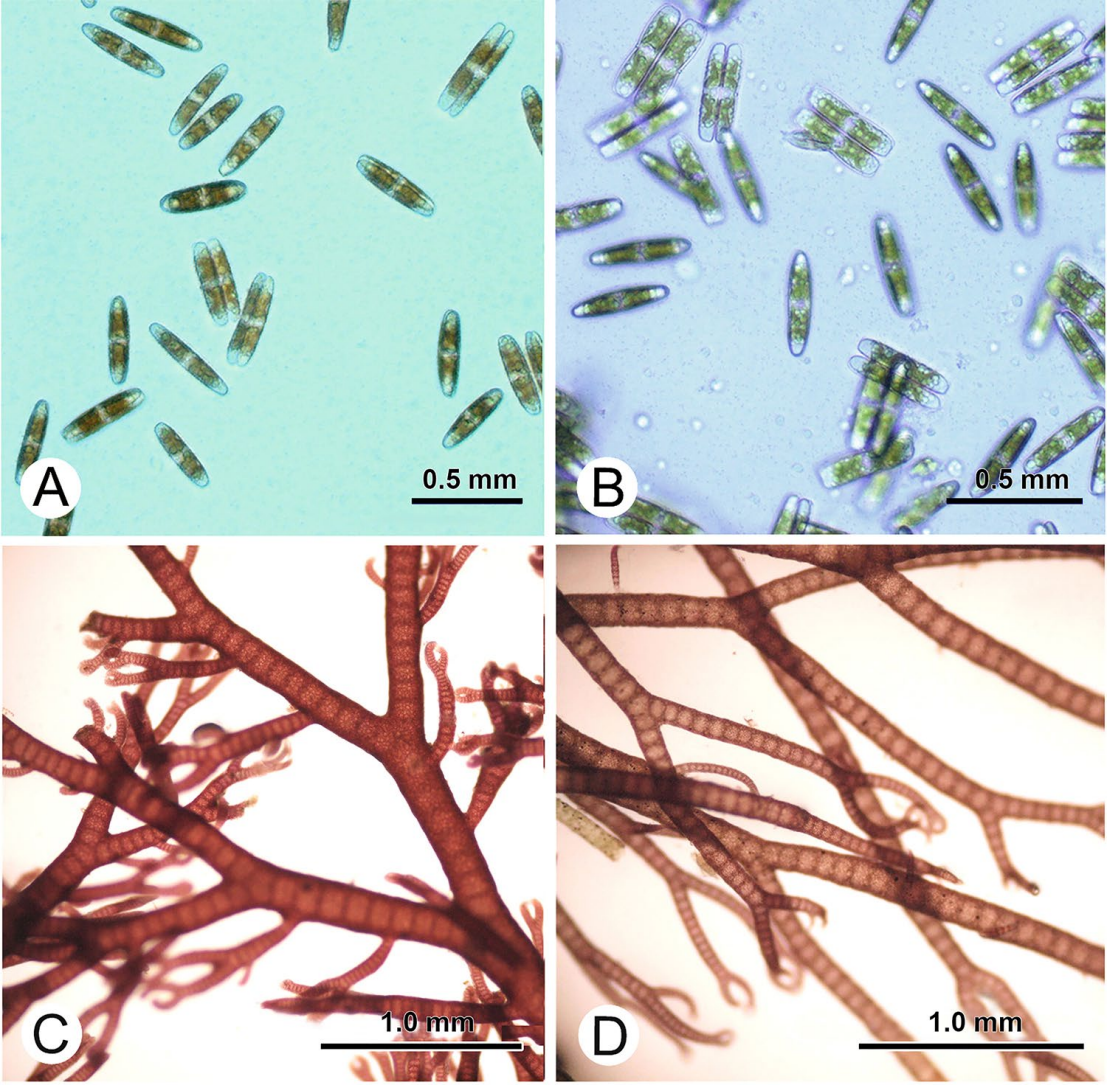
Micro-foulers and macro-foulers. **A** *Stauroneis* sp. from China; **B** *Stauroneis* sp. from Germany; **C** *Ceramium tenerrimum* from China; **D** *Ceramium virgatum* from Germany

Diatoms are very common settlers on living and non-living surfaces in marine environments and have repeatedly been used as model microfoulers in studies related with the chemical antifouling defence of invasive macroalgae (Plouguerne et al. 2008; Schwartz et al. 2017a, b). Wang et al. (2017a), in common garden experiments, compared the capacity of four native Asian and four invasive European populations of *A. vermiculophyllum* to repel two pennate diatom species of the genus *Stauroneis* (Fig. 2) that were both originally isolated from *A. vermiculophyllum*. One of the tested diatoms originated from a native habitat of the host in China, while the second originated from an invaded habitat in Germany. In bioassays both diatom species settled less on living host specimens originating from non-native than on specimens originating from native populations, with reductions by 72% and 50% for the Chinese and the German diatom species, respectively. In a second series of experiments, Wang et al. (2017a) also compared the effect of surface metabolites isolated from specimens of *A. vermiculophyllum* that had previously been acclimatized to a common garden. Dichloromethane (DCM)-extracted and hexane-extracted surface metabolites were impregnated on paper filters at their natural concentration and settlement of the same two diatom species on such filters was compared. Within 3 h, 8% and 9% less of the diatoms from China and Germany, respectively, attached to filters coated with extracts from non-native *A. vermiculophyllum* specimens, as compared to extracts from native *A. vermiculophyllum.* Diatoms from both habitats attached by 4% less to surfaces covered with hexane-extracted metabolites (non-polar compounds) than to those coated with DCM-extracted metabolites (polar compounds). The deterrent compounds were not identified, but non-polar surface metabolites probably played an important role for the deterrence of diatoms, and the observed difference between native and non-native host populations confirms that an upward-shift in the production and release of compounds that deter diatom settlers has occurred during the invasion history of *A. vermiculophyllum*.

Various *Ceramium* species are common macroalgal epiphytes on *Agarophyton* and other Gracilarioids (Fletcher 1995; Leonardi et al. 2006; Martín et al. 2013; Muñoz and Fotedar 2010). They are capable of secondary attachment and can directly penetrate into the host tissue by formation of hapteria (Leonardi et al. 2006; Lion et al. 2006; Michetti et al. 2016). The capacity of *Ceramium tenerrimum* (from Rongcheng, China) and *Ceramium virgatum* (from Kiel, Germany) (Fig. 2) to settle on living thalli of native and invasive *A. vermiculophyllum* within two weeks of colonization was compared by Wang et al. (2017a) using host specimens from four European and four Asian populations, that had been adapted to common garden conditions. On average, both *Ceramium* filaments attached by 33% less to invasive than to native *A. vermiculophyllum*. Further assays with surface metabolites isolated from the different populations were conducted in this study. On average, 10% less *Ceramium* filaments attached to surfaces that had been coated with extracts gained from non-native specimens at natural concentrations, compared to extracts from native *A. vermiculophyllum.* Fewer *Ceramium* filaments settled on extracts gained with DCM than on extracts gained with hexane. In this respect, the relative activity strength of metabolites gathered with DCM and hexane differed from that observed with diatom settlers (see previous section), which strongly suggests that the deterrent compounds affecting *Stauroneis* and *Ceramium* are not the same. It follows that the expression of different deterrent compounds that target *Stauroneis* and *Ceramium* has been similarly upregulated in non-native populations of *A. vermiculophyllum*, as compared to native populations of the same species.

## Evidence of shifting defence against fouling organisms

Fouling communities are usually composed of dozens or even hundreds of species in the same habitat (Saha and Wahl 2013). The majority of these organisms are generalists with respect to substratum choice and can settle on a variety of living organisms and non-living surfaces alike (Wahl 1997). Thus, when reaching a new environment, an invasive marine organism could potentially be subject to settlement of a wide range of different fouling organisms. Any comparative assessment of the overall defence capacity of organisms against fouling should, for this reason, not only be conducted under laboratory conditions and with selected foulers, but also in situ under field conditions. However, organisms originating from different ecoregions can usually not be directly exposed to fouling in the same environment, because such an approach would pose an inherent risk of introduction of new genetic material into this environment. Wang et al. (2017b) were able to overcome this problem for the first time. They enclosed *A. vermiculophyllum* specimens of identical size that originated from four native and four non-native populations, that had been previously acclimatized to the same common garden conditions, into transparent dialysis membrane tubes. The tubes were exposed to natural fouling both in the native (Akkeshi Bay, Japan) and the non-native (Kiel Fjord, Germany) range of the species. The tubes prevented the escape of genetic material into the environment but were diffusible for metabolites smaller than approximately 500 Da. Both in the native and non-native habitats, significantly fewer fouling organisms settled on the outer surface of dialysis tubes containing non-native *A. vermiculophyllum* individuals within three weeks than on tubes containing native individuals. Since all individuals were kept in dialysis membrane tubes that were only diffusible for small metabolites, the differences in settlement must have been due to differences in the release of chemical compounds by *A. vermiculophyllum* individuals belonging to different populations. These observations strongly suggest that the defensive capacity of *A. vermiculophyllum* against fouling organisms in general is higher in non-native populations (in Europe) than in native populations. Thus, also in this respect, a defensive upward shift has apparently occurred during *A. vermiculophyllum*’s invasion history.

Fouling pressure in the sea varies seasonally and the antifouling activities of macroalgae sometimes exhibit corresponding seasonal patterns (Hellio et al. 2004; Rickert et al. 2016; Saha and Wahl 2013; Wahl et al. 2010). Wang et al. (2018) reported that the antifouling capacity of *A. vermicullophyllum* also varied seasonally and correlated with fouling pressure in the Kiel Fjord, Germany. The abundance of foulers in that study was on average 14% lower on *A. vermiculophyllum* individuals than on the PVC panels, that were used as a non-living control. However, the abundance on both substrates changed seasonally, peaking in summer with the natural fouling pressure. DCM-based surface extracts of *A. vermiculophyllum* sampled from the Kiel Fjord during this period exhibited a corresponding pattern of deterrence of the epiphyte *C. tenuicorne* in bioassays (Wang et al. 2018), suggesting that *A. vermiculophyllum* can generally adjust its chemical defence capacity to demand.

## Evidence of shifting defence against bacterial settlers

The capacity of native and non-native *A. vermiculophyllum* populations to deter bacterial settlers has also been compared. Saha et al. (2016) isolated bacteria co-occurring with—but not settling directly on—*A. vermiculophyllum* from stones in the native (coasts of South Korea) and non-native distribution range (coasts of Denmark and Germany) of the alga. As in the previously mentioned studies, non-polar and relatively polar surface-associated metabolites of both native and invasive *A. vermiculophyllum* were extracted with a mixture of DCM and hexane 1:4 (v/v), coated at natural concentrations into microtiter well plates and compared for their capacity to deter the different bacterial isolates. In this study, both native and non-native *A. vermiculophyllum* populations proved to be equally well defended against presently co-occurring bacteria, isolated from their respective ranges, but both groups of populations also exhibited a reduced chemical defence capacity against bacteria from the other range: specimens from the native distribution range were relatively less well defended against bacteria from the non-native range, whereas specimens from the non-native range had apparently lost capacity to deter bacteria from the native range. At the same time, specimens from the native range were on average associated with three times more bacteria than specimen from the invaded range. Thus, the observations by Saha et al. (2016) suggest that the anti-bacterial defence of *A. vermiculophyllum* has also shifted, result not only in an increased defensive capacity against settlers in new habitats, but also in a loss of defensive capacity against former settlers in the old habitats.

However, the epiphytic bacterial communities associated with macroalgae may not only consist of detrimental organisms, but also of beneficial ones that are important to macroalgal development and health (Egan et al. 2013). Correspondingly, Saha and Weinberger (2019) reported that there are three types of epiphytic bacteria on the surface of native and invasive *A. vermiculophyllum*; pathogenic, beneficial and neutral. Pathogenic bacteria have the capacity to induce a bleaching symptom in *A. vermiculophyllum*, while beneficial bacteria have the capacity to prevent the induction of this symptom. Based upon analysis of 60 cultivatable bacterial isolates, that all originated from healthy specimens of *A. vermiculophyllum,* the authors estimated that approximately one-third of all surface-associated microbiota could be protective and thus beneficial, while approximately 5% could be facultative pathogens, which may only become virulent if the protective microbial component is weakened (Saha and Weinberger 2019). These authors further demonstrated that metabolites from the surface of *A. vermiculophyllum* (again extracted in DCM and hexane 1:4 (v/v) and tested at a onefold natural concentration) attracted beneficial strains but deterred pathogenic epiphytic bacteria. The bioactive metabolites were not identified and their exact source (host or specific bacterial taxa) is still unknown. However, *A. vermiculophyllum* and an important component of its surface microbiome apparently form a symbiosis-like association that can stabilize itself by excretion of probiotic and antibiotic compounds. Observations pointing into the same direction were also reported for *Fucus vesiculousus* (Lachnit et al. 2010) and *Delisea pulchra* (Longford et al. 2019), as well as for the rhizosphere of terrestrial plants, where specific bacteria can not only facilitate nutrient acquisition but also support plant growth under biotic and abiotic plant stress (Durán et al. 2018; Lareen et al. 2016).

The concept of a stable symbiotic relationship between *A. vermiculophyllum* and certain microbiota was further supported by a recent study that compared the microbial communities associated with this alga throughout its distribution range (Bonthond et al. 2020): A core set of 14 bacterial taxa was identified that was consistently present—either epibiotic or endobiotic or both—in all *A. vermiculophyllum* samples originating from the 14 populations throughout Asia, Europe and the North American east and west coasts. Moreover, a larger set of 290 additional taxa was present in specimens from all investigated populations, although sometimes absent in single samples. Apparently, *A. vermiculophyllum* has been accompanied by a selection of closely associated microbiota during its invasion history. Despite this pertaining association with a core microbiome, host and associated microbiome together obviously underwent shifts in their defence capacity during the invasion process, as indicated by the different capacities for deterrence of microbial settlers in native and invasive *A. vermiculophyllum* that were summarized above.

## Conclusions and future research direction

**Fig. 3:**
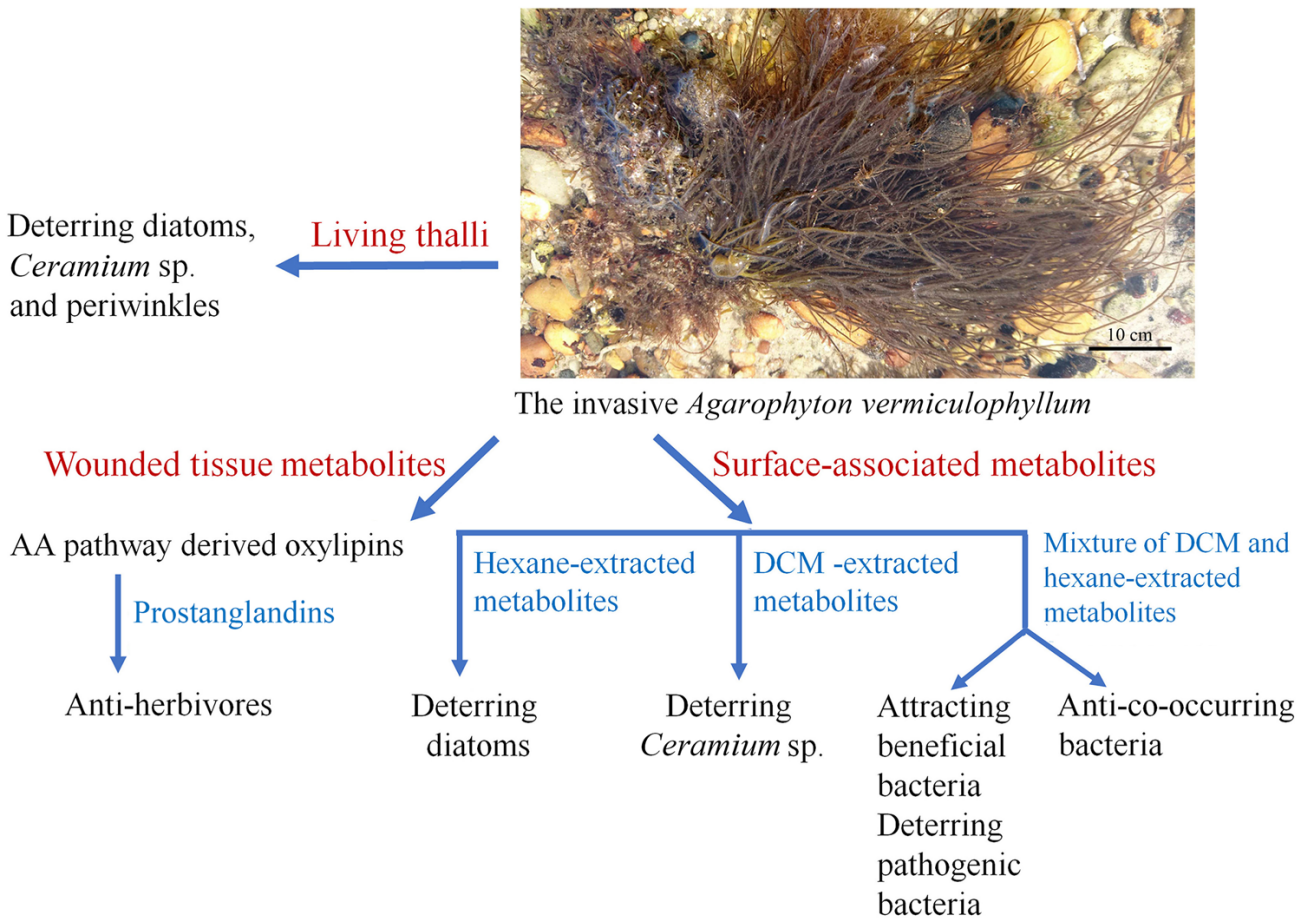
Summary of the results related to the chemical defence in the invasive *A. vermiculophyllum*. Surface-associated metabolites, wound-activated metabolites and living thalli-mediated chemical defences are identified in the invasive *A. vermiculophyllum* by comparative studies with native populations. *AA* arachidonic acid, *DCM* dichloromethane

Taking all the evidence outlined above together, *A. vermiculophyllum* clearly underwent multiple shifts in its defence behavior when (or after) it invaded new habitats. Non-native populations generally exhibited stronger defences against periwinkle consumers, epiphytic *Ceramium*, diatom settlers of the genus *Stauroneis* and macrofoulers than native populations. Non-native populations were also better defended against bacterial settlers in new environments and only in the case of amphipod consumers evidence of shifting defence was not detected. Thus, as in some other study models—not tested in common gardens, but with field material (e.g., Schwartz et al. 2017a, b; Wikström et al. 2006)—the case of *A. vermiculophyllum* supports the validity of the SDH for macroalgal invaders and the more general idea that the invasion success of seaweeds is affected by biotic interactions in the newly gained environments. Shifting defence may have facilitated—and may have even been a necessity—for the rapid global expansion of *A. vermiculophyllum* into new ranges (Fig. 3). However, multiple different shifts can be differentiated in this model organism. In the case of anti-periwinkle defence, wound-activated production of eicosapentaenoids was identified as the underlying mechanism. The same mechanism could potentially also contribute to *A. vermiculophyllum*’s defence against *Ceramium*, which causes cell disruption when it penetrates into the host thallus (Leonardi et al. 2006). However, wounding is rarely—if ever—observed when diatoms such as *Stauroneis* or bacteria settle on the surface of seaweeds. This circumstance strongly suggests that other compounds than eicosatetraenoids must drive the deterrence of micro-settlers by *A. vermiculophyllum*. Thus, upregulation of not only one, but multiple defence-related metabolic pathways was apparently selected during the invasion history of this alga. This view is also supported by the observation that metabolites with activity against different groups of settlers could be best extracted from the algal surface with different solvents (in some cases hexane, in some cases DCM, see Fig. 2). The active compounds against epibionts in *A. vermiculophyllum* have not yet been identified. However, as most epibionts—including *Ceramium* and *Stauroneis*—are substrate generalists, raises the expectation that the defensive shift in *A. vermiculophyllum* against eukaryotic foulers may be based upon an upregulation of broad-spectrum defence metabolites, rather than “novel weapons”, i.e., deterrence of settlers with specialized narrow-spectrum defence metabolites. A different case is the defence of *A. vermiculophyllum* against bacterial settlers. Because non-native populations gained defensive capacity against new enemies but lost defensive capacity against old enemies, this could hint at more specialized chemical defences, which would then support the NWH.

Common garden studies with more seaweed models would be required to further substantiate the view that shifting defence may happen frequently in seaweeds. Interestingly, the few studies that have so far been conducted reported, in most cases, evidence of shifting defence, while most of the (larger number) studies that tested the NWH found no support for it in seaweed models. This could suggest that adaptive responses on the side of the host are more frequent than invasion success due to novel weapons. However, to test the SDH further, it would again be necessary to directly compare native and non-native populations. Where common garden experiments are impossible, an alternative (although less rigorous approach) might be to compare field-samples from native and non-native populations, collected in both ranges over broad geographic and climatic scales. Such sets of on-site conserved samples could be analyzed for their content in defense compounds or for expression patterns of identified defense genes.

However, a bottleneck for the testing of defense hypotheses in marine organisms is the still relatively limited knowledge of relevant defence compounds, mechanisms and genes, as also exemplified above for *A. vermiculophyllum*. There is an urgent need to identify metabolites that are responsible for the defences of seaweeds against various settlers, consumers and pathogens and to identify the metabolic pathways involved in the production of these compounds. Ongoing bioprospection can be expected to advance our knowledge of bioactive compounds in a larger spectrum of invasive and non-invasive seaweeds relatively soon. However, marine ecologists will still need to demonstrate the ecological relevance of identified compounds (i.e., their deterrent effects on enemies).

A full understanding of the defence systems and biotic interactions of seaweed invaders in new habitats may facilitate the development of community management strategies for the mitigation of their further long term expansion. Marine bioinvasions—not only of seaweed, but also of animals and other organisms—have become one of the growing global concerns and have multiple negative ecological consequences, causing biodiversity loss in the new ranges. Such global problems cannot be exclusively solved on a local scale. Researchers skilled in ecology, microbiology, immunology, molecular genetics and distribution modeling should take efforts to establish more international collaborations to promote significant progress in the field of marine invasion ecology.
